# Simple model systems reveal conserved mechanisms of Alzheimer’s disease and related tauopathies

**DOI:** 10.1186/s13024-023-00664-x

**Published:** 2023-11-10

**Authors:** Yuwei Jiang, Lesley T. MacNeil

**Affiliations:** 1 Department of Biochemistry and Biomedical Sciences, McMaster University, Hamilton, Canada; 2 Farncombe Family Digestive Health Research Institute, McMaster University, Hamilton, Canada; 3 Michael G. DeGroote Institute for Infectious Disease Research, McMaster University, 1280 Main St W, Hamilton, ON L8S 4K1 Canada

**Keywords:** Neurodegeneration, Aggregation, Alzheimer’s disease, Tauopathy, Amyloid-β, Genetic modifiers, Cellular pathways, *S. cerevisiae*, *C. elegans*, *Drosophila*, Tau

## Abstract

The lack of effective therapies that slow the progression of Alzheimer’s disease (AD) and related tauopathies highlights the need for a more comprehensive understanding of the fundamental cellular mechanisms underlying these diseases. Model organisms, including yeast, worms, and flies, provide simple systems with which to investigate the mechanisms of disease. The evolutionary conservation of cellular pathways regulating proteostasis and stress response in these organisms facilitates the study of genetic factors that contribute to, or protect against, neurodegeneration. Here, we review genetic modifiers of neurodegeneration and related cellular pathways identified in the budding yeast *Saccharomyces cerevisiae*, the nematode *Caenorhabditis elegans*, and the fruit fly *Drosophila melanogaster*, focusing on models of AD and related tauopathies. We further address the potential of simple model systems to better understand the fundamental mechanisms that lead to AD and other neurodegenerative disorders.

**Supplementary Information:**

The online version contains supplementary material available at 10.1186/s13024-023-00664-x.

## Background

Alzheimer’s disease (AD) is the most common form of dementia. It is characterized by the accumulation of amyloid-beta in senile plaques and hyperphosphorylated Tau in neurofibrillary tangles (NFTs) [22]. Mutations in the amyloid precursor protein (APP) or the proteins that cleave it, the presenillins, PSEN1 and PSEN2 (as part of the gamma secretase), have been identified in familial AD [325]. Familial cases are of early onset and account for only a small percentage of AD cases. Most cases of AD are considered sporadic, but many genes associated with altered disease risk have been identified [151].

AD begins with a preclinical stage where no symptoms are evident but amyloid plaques and neurofibrillary tangles are present in the brain. Mild or early stage AD is characterized by loss of concentration, disorientation, and mood changes that occur due to pathological changes in the cortex and hippocampus. The moderate stage is associated with increased memory loss, difficulty in reading, writing, and speaking as neurons in the cerebral cortex begin to degenerate. In late-stage, patients suffer from severe cognitive decline and motor impairments [252]. Tau pathology is well correlated to disease severity. Tau pathology begins in the transentorhinal cortex (Braak stage I). As the disease progresses, Tau pathology spreads to the hippocampus (Braak stage II/III) and later to regions of the cerebral cortex (Braak stage IV/V) [21]. Tau modification is also an important predictor of degeneration, abnormal Tau phosphorylation is observed before the formation of neurofibrillary tangles.

Models of AD and other tauopathies have been developed in many animals, including rodents, primates, and simple model organisms [4, 69, 108, 144, 269]. In addition, mammalian cell culture and yeast models have been used to study molecular events that contribute to Tau and Aβ toxicity. However, no single AD model is ideal for all questions. Non-human primates most closely mimic human biology with a well-developed prefrontal cortex and development of age-associated senile plaques but due to cost and ethical considerations, are not suited to discovery-based research. Mouse models are widely used in AD research and have provided important insights. They can recapitulate phenotypes in relevant brain areas that cannot be studied in simpler organisms or cell culture models. However, it is a challenge to perform large-scale unbiased genetic or chemical screens in mice. Simpler models are more amenable to this type of analysis. Here we review genetic modifiers of Aβ and Tau-based AD models,we restrict our discussion to three organisms in which large-scale screens have been accomplished, *S. cerevisiae*, *C. elegans*, and *D. melanogaster*.

Simple model organisms offer several advantages for studying neurodegenerative diseases, including low-cost, unbiased high-throughput screening capabilities, ease of genetic manipulation, and availability of resources such as mutant and transgenic strains. Importantly, many human disease-associated genes have orthologs in these organisms [225]. 62% of human disease genes are conserved in flies [89]. At least 42% of human disease genes have a *C. elegans* orthologue [53] and 83% of the *C. elegans* proteome has a human homolog [171]. These organisms have orthologs of many neurodegenerative disease-associated genes, and cellular processes associated with neurodegeneration are largely conserved. Furthermore, expressing human disease-associated proteins in these models recapitulates many features of neurodegenerative disease [32, 42, 167].

While no single model can recapitulate all aspects of human disease, each model brings unique strengths that can provide important insights. The baker’s yeast *S. cerevisiae* is a powerful tool for studying gene function and genetic interactions. In addition to its ease of manipulation, *S. cerevisiae* has the most complete gene deletion collection of any eukaryote. In addition, available genome-scale protein–protein and genetic interaction datasets provide the ability to gain a more global view of gene function [50, 105, 268, 291, 330].

*Drosophila* and *C. elegans* have easy-to-visualize neurons and short lifespans suitable for aging studies. The simple neuroanatomy of *C. elegans*, together with its transparency, allows individual neurons to be studied in their correct biological context [2, 76]. The fruit fly *Drosophila* has a more complex nervous system [281] with more than 200,000 neurons, and a simple brain that can support complex behaviours [91]. Behavioural assays in *C. elegans* and *Drosophila* allow the assessment of neuronal function and the identification of dysfunction that precedes physical signs of neurodegeneration.

This review summarizes the genetic modifiers and evolutionarily conserved cell signaling pathways identified from high-throughput screens and targeted studies in *S. cerevisiae*, *C. elegans*, and *Drosophila* models of AD and related tauopathies. Similar analyses found overlap in genetic modifiers between these species and between models of different neurodegenerative diseases [40], van Ham et al. 2009). In this study, we focus specifically on models of AD and provide an updated list of reported genetic modifiers that includes 1,000 yeast genes, 176 *C. elegans* genes, and 953 *Drosophila* genes (Tables S1, S2, S3). These modifiers are associated with key cellular processes including protein synthesis, proteostasis, trafficking, mitochondrial function, cytoskeletal regulation, metabolism, cell signaling, and immune response.

## Main text

### Simple models of Alzheimer’s disease

The main defining pathological features of AD are the accumulation of amyloid-β (Aβ), a proteolytic product of the amyloid precursor protein (APP), and the aggregation of the microtubule-associated protein Tau (MAPT) [18]. Abnormal Tau aggregation is also observed in other neurodegenerative diseases, including Pick’s disease, progressive supranuclear palsy and frontotemporal dementia, collectively known as tauopathies [162, 232]. Despite extensive research, the function of Aβ, how its accumulation promotes neurodegeneration, and the link between Aβ and Tau aggregation, are not fully understood [158, 270].

Loss of function mutations in the *C. elegans* and *Drosophila* orthologs of *APP* and *MAPT* are viable, enabling the investigation of conserved functions of these genes [25, 46, 80, 132]. However, APP processing differs among these organisms. In mammals, the Aβ peptide is produced by proteolytic processing of the amyloid precursor protein (APP) by β and γ secretases. APP cleavage produces different forms of Aβ, including the majority species Aβ_40_ and the more toxic species Aβ_42_ [335]. Aβ_42_ peptides, which account for approximately 10% of total Aβ produced, are more prone to aggregation [24]. An increased Aβ42/40 ratio is observed in familial AD, suggesting a crucial role for Aβ_42_ in disease pathogenesis [7, 267]. Moreover, an elevated ratio of Aβ42/40 induces Tau aggregation in cultured neuronal cells [169].

*C. elegans* and *Drosophila* lack a BACE homolog, the β-secretase that cleaves APP and the amyloid-precursor-protein-like (APL-1 of APPL) proteins shows little homology to the human protein in the Aβ region, suggesting they do not produce an Aβ equivalent. However, *Drosophila* APPL is processed by secretases to form secreted fragments, an Aβ-like peptide, and an intracellular fragment [35]. This processing is similar to the human protein, but the order of the cleavage sites on the protein is reversed. Interestingly, although APPL does not contain the human Aβ-like sequence, APPL can be cleaved by a fly β-secretase-like enzyme to form neurotoxic peptides that aggregate into amyloid deposits, suggesting that APPL may produce a structurally similar peptide [32].

The lack of conservation in the cleavage of amyloid precursor proteins precludes the use of the full-length human disease-associated APP transgenes as a model for AD. The expression of a secreted human Aβ_42_ peptide is therefore used to overcome this challenge [51, 139, 182]. While this approach may limit our ability to study some aspects of AD biology, including the regulation of APP expression and APP cleavage, modifiers with known association to AD have been identified in genetic screens using these models.

Yeast models have been used to study the oligomerization, aggregation, and toxicity of Aβ [9]. In yeast, cytoplasmic or ER-targeted Aβ_42_ has been used to model different aspects of Aβ toxicity. When Aβ is targeted to the ER, it progresses through the secretory pathway but is retained by the yeast cell wall, allowing it to interact with the plasma membrane and endocytic machinery [297]. Both ER-targeted and cytoplasmically expressed Aβ reduce growth rate, a phenotype that has been used to identify suppressors and enhancers of Aβ-associated toxicity [27, 55, 297]. Yeast, as single-cell eukaryotes, have the advantage of simplicity in studying cell-autonomous functions, however, they cannot recapitulate neuron-specific processes and organismal responses that may be critical in the development of neurodegeneration.

In *C. elegans,* the secretion of Aβ_42_ from muscle leads to paralysis [64, 82, 182]. This model has been extensively used because it provides an easy-to-score phenotype and is amenable to large-scale screening [154]. Pan-neuronal expression of Aβ_42_ has also been used in *C. elegans*. These animals have defects in chemotaxis, behavioural responses, and movement but do not produce the dramatic paralysis phenotype observed with muscle-specific expression [63, 277]. Large-scale RNAi screens have been performed with both models, with only one gene, the HSP70 family member *hsp-1*, found in both screens [154, 165].

Similar to other models, expression of AD-related proteins in flies results in aggregation and impaired cellular functions. Expressing hAPP in flies, together with the cleaving enzyme BACE and presenilins, in photoreceptor cells generates amyloid plaques and leads to neurodegeneration [110]. In addition, ubiquitous expression of these genes caused ectopic wing vein formation and a shortened lifespan. Amyloid deposition and neurodegeneration were also observed when human Aβ_42_ was expressed in the fly brain [84, 138]. Interestingly, expressing Aβ_40_ causes age-dependent learning defects but no obvious neurodegeneration, consistent with observations in mammalian systems where Aβ_40_ is less fibrillogenic and toxic than Aβ_42_ [139, 216].

### Tau-based models of disease

Tau is a highly soluble protein that binds tubulins and promotes the assembly and stabilization of microtubules. In the human brain, six major Tau isoforms are generated by alternative splicing [104]. Isoforms have one (1N), two (2N) or no amino-terminal inserts (0N) and differ in the exclusion or inclusion of exon 10, resulting in a protein with either 3 (3R) or 4 (4R) microtubule-binding regions. In a normal adult brain, the ratio of 3R to 4R isoforms is approximately equal [124]. Both 3R and 4R isoforms are found in the AD brain but the ratio of the two may change during the course of the disease [124]. The phosphorylation status of Tau also changes in AD. Hyperphosphorylation of Tau precedes its aggregation into NFTs [3]. Although *MAPT* mutations are not a cause of AD, they are causal in other tauopathies including frontotemporal dementia (FTD) and Pick’s disease [141].

**Fig. 1:**
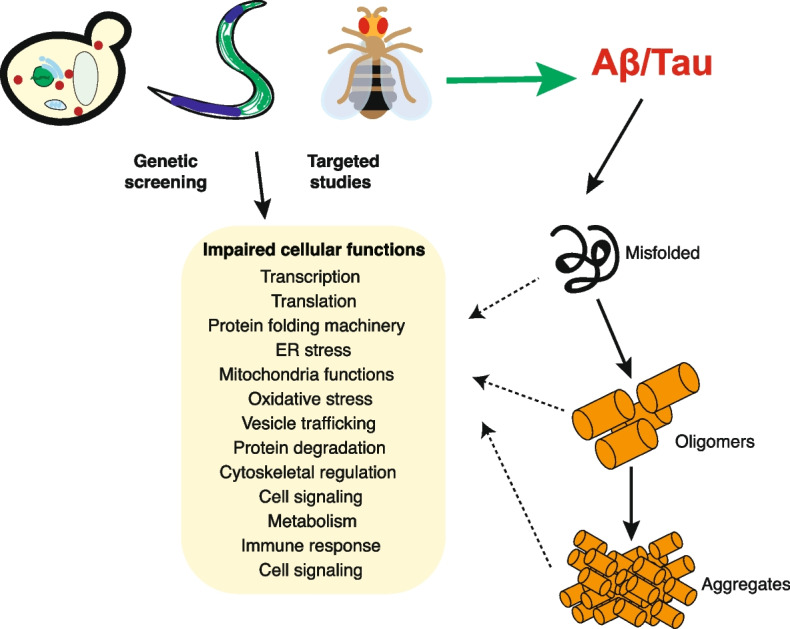
Modeling Alzheimer's disease in simple model systems. Expressing AD-related proteins Tau and Amyloid beta, in simple model systems, including *S. cerevisiae, C. elegans, and Drosophila melanogaster* recapitulates many disease-associated phenotypes

Expression of FTD-associated Tau variants (V337M, V301L, R406W) has been used to model AD in both flies and worms [2, 107, 204, 245]. Expression of these Tau variants in *C. elegans* induces disease-associated pathologies, including phosphorylation of Tau at disease-relevant sites, accumulation of insoluble Tau aggregates, synaptic loss, decreased neuronal function and neurodegeneration [23, 167, 209]. In studies comparing wild-type and mutant Tau, disease-associated mutations produced more severe phenotypes [167]. Transgenic flies expressing mutant human Tau show increased Tau phosphorylation and disease-associated Tau conformations, reduced lifespan, and vacuolization and degeneration of cortical cells [243, 274, 320, 321]. In *C. elegans*, impaired locomotion occurs before insoluble Tau aggregates are detected, suggesting that neurodegeneration in this model is not a general effect of aggregated protein [167]. Increased Tau phosphorylation and age-related neurodegeneration were observed in a *Drosophila* model in the absence of neurofibrillary tangles [320, 321]. Overall, expressing AD-related proteins in simple models recapitulates many, but not all, features of the disease (Fig. 1) and provides insight into the progression of the disease.

**Fig. 2:**
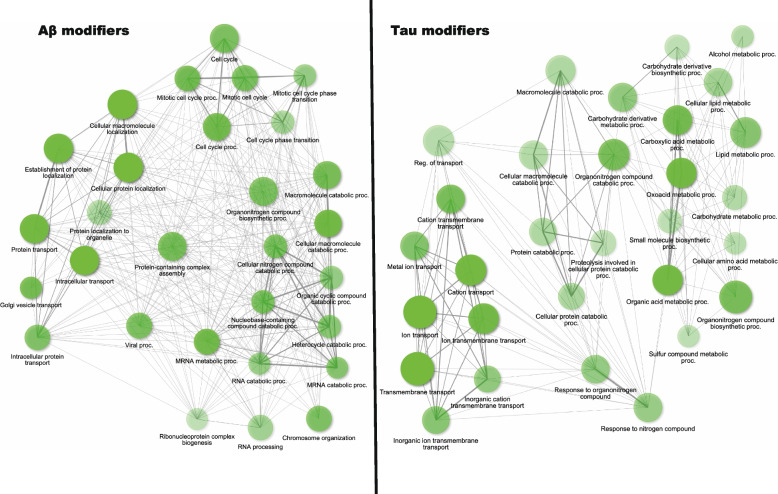
Pathways enriched amoung genetic modifiers of AD models. Human homologs of genetic modifiers were analyzed using ShinyGO v0.77 (Ge et al. 2020). Nodes represent the top 30 enriched GO terms. Node size is scaled to the number of genes. Darker nodes are more significantly enriched. Edge thickness is proportional to the number of overlapping genes in each category

Large-scale screens in yeast, worms, and fruit flies have identified many genetic modifiers of amyloid-beta and Tau toxicity (Table 1) [17, 30, 42, 93, 154, 156, 165, 255, 274, 275, 285, 297]. Here we summarize these modifiers and compare findings across species. Many of these modifier genes can be linked to cellular pathways and processes associated with AD, such as protein trafficking and localization, cell cycle, metabolic processes, gene expression, and stress response (Fig. 2).

**Table 1:** Genetic modifier screens in *S. cerevisiae*, *C. elegans* and *D. melanogaster* models of Alzheimer’s disease

Organism	Transgene	Screen	Phenotype	Reference
*S. cerevisiae*	secretory GAL-α-prepro- Aβ_42-_GFP	yeast deletion collection of ∼6000 ORFs	growth	[93]
*S. cerevisiae*	secretory GAL-Kar2- Aβ_42_	overexpression library of 5532 full-length ORFs	growth	[297]
*S. cerevisiae*	secretory GPD-Kar2- Aβ_42_	~ 4300 deletion and ~ 1200 temperature sensitive mutant strains	growth	[42]
*C. elegans*	*myo-3p:*:Aβ_42_ (muscle)	RNAi against 7970 *C. elegans* genes with human homologs	paralysis	[154]
*C. elegans*	*aex3*::hTau V337M (pan neuronal)	RNAi against 16,757 genes	uncoordinated (Unc) locomotion	[165]
*Drosophila*	Aβ_42_ expressed in the eye	1963 EP insertions^a^	rough eye phenotype	[30]
*Drosophila*	Aβ_42_ expressed in central nervous system	3000 GS insertions^a^	longevity	[255]
*Drosophila*	hTauV337M expressed in the eye	2276 EP insertions	rough eye phenotype	[274]
*Drosophila*	hTauV337M expressed in the eye	RNAi sequences for 87 fly homologs of human candidate genes	rough eye phenotype	[275]
*Drosophila*	hTau expressed in the eye	RNAi sequences for 74 fly homologs of human candidate genes	quantification of eye size	[66]
*Drosophila*	hTau expressed in the eye	144 *Drosophila* miRNAs	quantification of eye size	[285]
*Drosophila*	hTauV337M expressed in the eye	1250 P{Mae-UAS.6.11} insertion^a^	rough eye phenotype	[17]
*Drosophila*	hTau expressed in the eye	RNAi sequences targeting 2,645 *Drosophila* genes	rough eye phenotype	[156]

^a^EP, GS (Gene Search), and P{Mae-UAS.6.11} lines contain gene insertions that allow forced expression of genes using the GAL4-UAS system, they typically result in over or mis-expression of the associated gene but can also result in loss of function

While extensive validation of many of these modifiers is lacking, modifiers identified in different studies could reveal important regulators of neurodegeneration and identify processes that are important for maintaining neuronal health. We performed functional annotation of modifiers from each species using online tools including FunSpec, GO Slim Mapper, Wormcat, Flybase, and GLAD [20, 120, 130, 135, 257] (Tables S6-S11). We also identified human homologs of these genes (Tables S1-S5). Homologs of AD-associated genes reported from genome-wide association studies (GWAS) were identified by comparing our list to the > 900 loci collected in the Alzheimer’s Disease Variant Portal (ADVP) [168]. In *Drosophila,* 60 genes (6%) identified as modifiers had potential human orthologs with some association to AD. Of the 176 modifiers identified in *C. elegans*, 23 genes had no predicted human ortholog and seven genes (4%) had orthologs listed in the ADVP database (Supplementary tables).

### AD-associated pathways in simple model systems

**Fig. 3:**
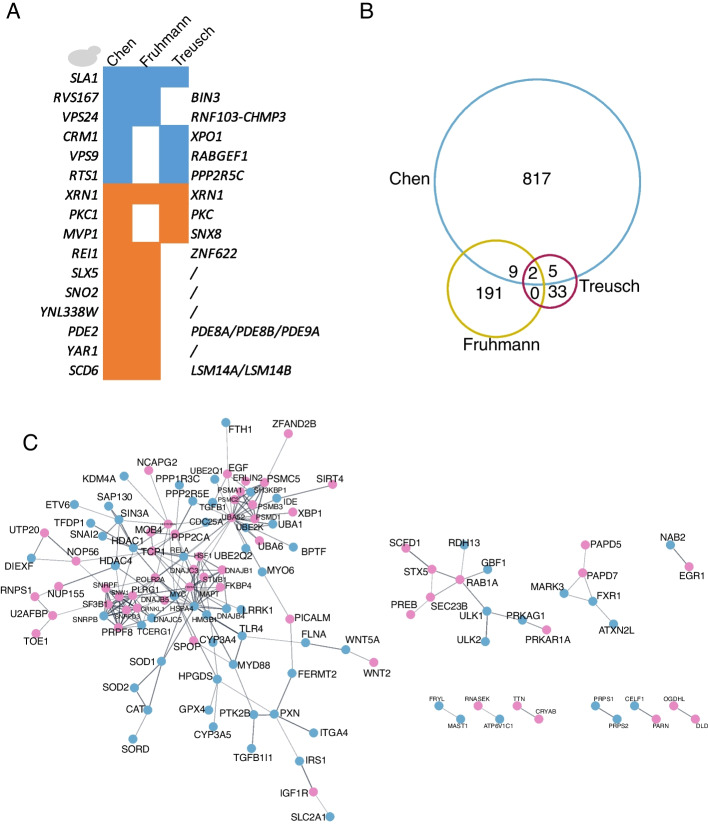
Enhancer and suppressor screens identify distinct and overlapping gene sets within, and between species. **A** Summary of three large-scale screens performed in *S. cerevisiae*. Human orthologs are shown on the right. Blue colour indicates that the gene function was protective, orange indicates gene function enhances toxicity. **B** Venn diagram showing overlap of genes recovered between yeast screens. **C** Physical interaction network of human orthologs of genes identified in *C. elegans* (pink) or *D. melanogaster* (blue) as modifiers of AD models. Network from Biogrid, 0.4 confidence, physical interaction subnetwork

The largest collection of modifiers of Aβ toxicity was identified in yeast. Three separate Aβ modifier screens were carried out, two using loss-of-function approaches and one using an overexpression system [42, 93, 297]. Although over 1000 genes (~ 17% of the genome) were identified as modifiers, only 16 genes had the same effect in more than one screen and only two genes (XRN1 and SLA1) had the same effect in all three screens (Fig. 3A &B). Twelve of these genes had human orthologs, with two (PDE2 and SCD6) identified as potential risk genes for AD [1]. PDE2 is a phosphodiesterase that enhances Aβ toxicity in yeast. It is homologous to several human phosphodiesterases, including PDE9A, which regulates cGMP and functions in learning and long-term memory. Consistent with PDE9A promoting neurodegeneration, PDE9A inhibitors have had success in pre-clinical studies where they improve cognitive function in rats [305]. SCD6, an ortholog of the processing body assembly factor LSM14A, functions in RNA processing.

The lack of overlap in yeast screens could reflect a limitation of the phenotype used to score toxicity in yeast. Because the yeast screens use growth as a readout, there is potential for false positives to arise when deletions affect growth. While mutants with growth defects are generally either removed from consideration or normalized in some way, there is the potential for Aβ to act as a sensitized background, producing synthetic interactions that are not specific to Aβ biology. Further analysis would be required to confirm the role of these homologs in AD-related processes.

**Fig. 4:**
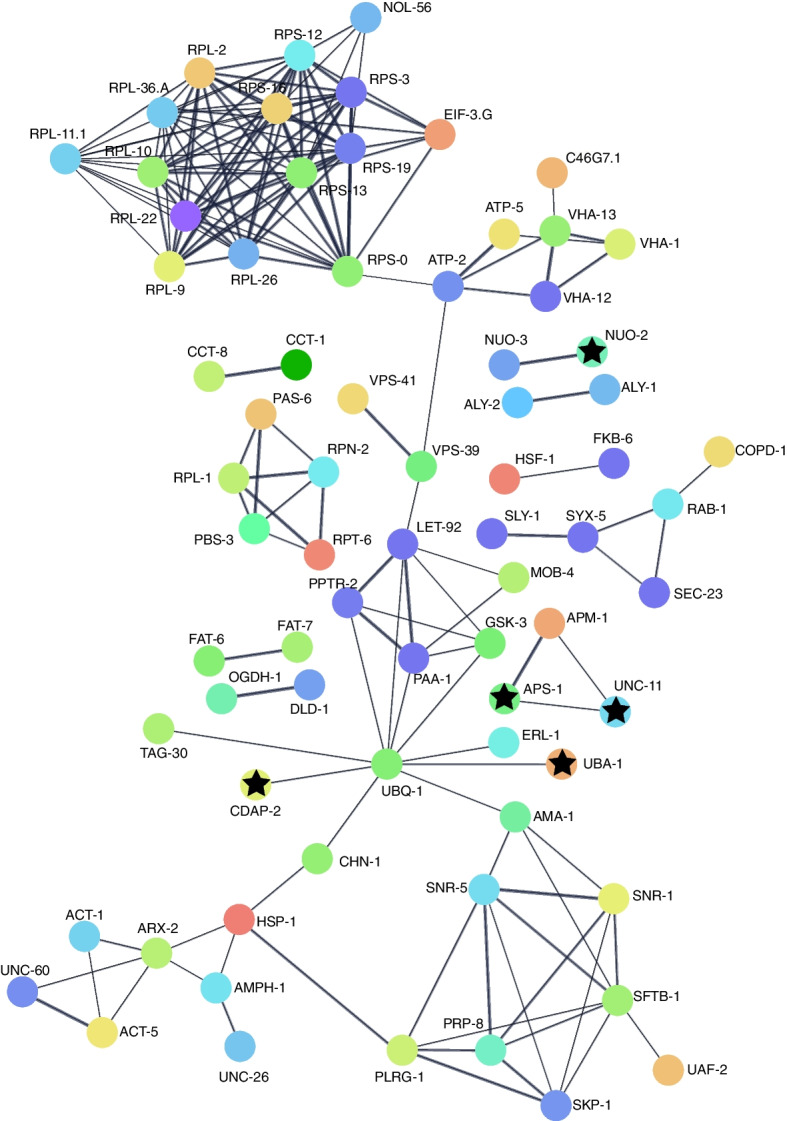
*C. elegans* interaction network. Physical subnetwork generated by String. Stars indicate genes with human orthologs with some evidence of AD involvement (Alzheimer's disease variant portal). Weight of edges indicates confidence score. Genes that did not interact with another gene in the network were removed

**Fig. 5:**
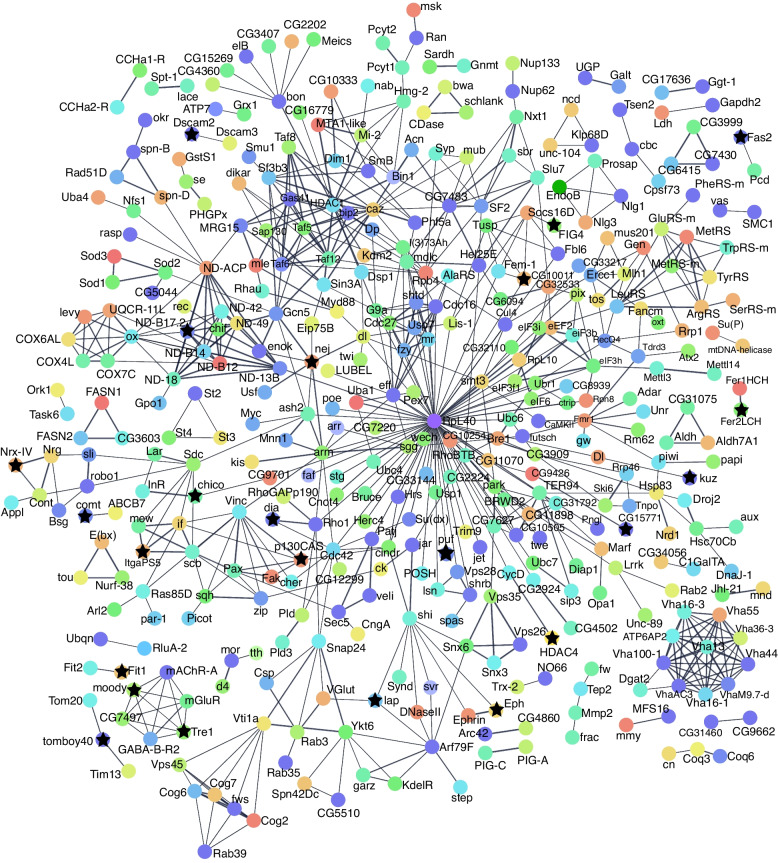
Drosophila interaction network. Physical subnetwork of modifier genes generated by String. Stars indicate genes with human orthologs with some evidence of AD involvement (Alzheimer's disease variant portal). Weight of edges indicates confidence score. Genes that did not interact with another gene in the network were removed

Similar to what was observed in yeast, a comparison of the *C. elegans* and *Drosophila* screens showed little overlap. No overlap was found in *Drosophila* and *C. elegans* Aβ screens, and only one gene (*fat-7*/CG8630), a homolog of human *SCD/SCD5*, had the same effect in Tau screens across species. This may be attributed to differences in cell type expression or phenotypic output. In the two largest *C. elegans* Aβ screens, Aβ was expressed in different compartments (one neuronal and one muscular) and different phenotypic readouts were used. While these screens have little overlap between species on a gene-by-gene basis, they do overlap in the types of genes and processes that are recovered. Primarily, these genes can be categorized into some common functional groups already associated with neurodegeneration, including proteostasis, trafficking, cellular stress-related pathways including ER stress and oxidative stress, immune response, metabolism, cytoskeleton, and signaling (Fig. 2). We also found that physical interactions had been reported between some of the modifiers (Figs. 4 and 5). Additionally, we examined the reported protein interactions for the human orthologs of *C. elegans* and *Drosophila* modifiers and found that they could be connected in a larger network (Fig. 3C).

### Dysregulation of protein folding machinery promotes AD

The misfolding and aggregating of proteins are countered by the action of molecular chaperones that support proteostasis by promoting proper folding or by promoting the destruction of aberrant proteins by the ubiquitin-proteosome or autophagy systems [261]. Although chaperones would generally be expected to play a positive role in protecting against neurodegeneration by promoting proteostasis, chaperones may also have negative effects by inadvertently stabilizing more toxic protein forms at the expence of less toxic ones [258].

Chaperones and co-chaperones were identified as modifiers of toxicity in large scale screens in yeast, *Drosophila* and *C. elegans*. Chaperones have been implicated in the response to protein aggregation associated with neurodegenerative diseases [258]. Increased expression of Heat shock proteins (HSPs) is observed in patients with AD and other neurodegenerative diseases [173]. Further, plasma Hsp70 levels are negatively correlated with cognitive performance in the elderly [278].

Many HSPs are protective in mammalian models of AD, with Hsp27, Hsp60, Hsp70, and Hsp90 decreasing protein aggregation and toxicity, or promoting neuroprotection [13, 16, 29, 133, 193, 195, 264]. Consistent with this, expression of the human Hsp70, either cytoplasmically or extracellularly, rescues memory decline in flies expressing Aβ_42_ [198] and Tau toxicity in *C. elegans* [209]. In a *Drosophila* Aβ model, a gain-of-function mutation in the Hsp70 co-chaperone Hsp110 extends lifespan [255]. High-throughput screens have identified a number of chaperones and co-chaperones that play protective roles in Aβ, Tau and other models of neurodegenerative disease [40].

The cochaperone CHIP that marks Hsp70 and Hsp90 substrates for degradation, ubiquitinates Tau for degradation [242]. As previously observed, CHIP interacts physically or mechanistically with a number of other modifiers of neurodegeneration [40]. In *Drosophila*, the deubiquitinase USP7 reduces Tau ubiquitination and promotes neurodegeneration, countering the activity of CHIP [156]. Knockdown of two E3 ubiquitin ligases that destabilize CHIP, RNF130 and RNF149, decreased neurodegeneration in *Drosophila* [156]. Knockdown of these genes in mice also reduced pathological Tau species and improved learning and memory in a tauopathy model [156]. Together, these studies suggest a conserved role for CHIP in preventing neurodegeneration.

Although it is likely that chaperones have wide-ranging effects on proteostasis, there is evidence to suggest that some chaperones act directly on Aβ or Tau. Analysis of *C. elegans* Aβ deposits identified six chaperones that co-immunoprecipitated with Aβ, including three alpha B-crystallin-related small heat shock protein HSP-16 s (HSP-16.1, HSP-16.2, HSP-16.48), two members of the Hsp70 family (C15H9.6 and F26D10.3), and a putative ortholog of a small glutamine-rich tetratricopeptide repeat-containing protein (SGT) (R05F9.10) [87]. Like Hsp70, overexpression of HSP-16.2 suppresses Aβ toxicity in *C. elegans* [88, 324]. Hsp90 physically interacts with Tau to aid in at least two functions (1) the interaction of Tau with microtubules and (2) the targeting of Tau for proteosomal degradation [150]. However, the unfolding of Tau mediated by Hsp90 also permits the formation of oligomers, which could promote toxicity [317].

In yeast (Ydj1), *Drosophila* (Droj2) and *C. elegans* (*dnj-7*), DNAJ proteins were identified in screens as enhancers of Aβ toxicity [42, 154, 254]. The yeast HSP40 family chaperone Ydj1, and its human ortholog DnaJA1, physically interact with Aβ and increase its accumulation in the mitochondria [254, 288]. This effect may be mediated through the ability of Ydj1 to delay Aβ fibrillization in favour of more toxic oligomers that are more easily transported into the mitochondria [254]. In contrast to its effects in the Aβ model, Droj2 downregulation enhanced Tau phenotype, suggesting different roles in regulating Aβ and Tau toxicity [156]. Although this requires further validation, it may reflect the fact that the effects in the Aβ model are independent of HSP70.

HSPA8/Hsc70 works in complex with a DNAJ protein DNAJB1, and Hsp110 as a disaggregase, which can aid in the clearance of amyloids [15, 72, 338]. Hsc70 and DNAJ proteins can also control the extracellular release of Tau [86]. In *Drosophila*, Hsc70Cb/Hsp110 and a co-chaperone Csp, were identified in two independent Tau-based screens, one using mutant and one using overexpression of wild-type Tau. In both cases, the expression of these proteins was detrimental [17, 156]. However, because Hsc70 proteins interact with a large number of proteins, it is difficult to identify the targets that are relevant to degeneration.

### The role of ribosomal proteins in AD

Ribosome dysfunction is an early event in the development of AD [62]. Both pathological (mutant or wild-type but hyperphosphorylated) and non-pathological Tau can associate with ribosomal proteins (RPs), with a different complement of proteins interacting with each [114, 160, 206]. In vitro studies suggest that, for pathological Tau, this interaction is inhibitory for translation [206].

Translation may also be inhibited through the regulation of ribosomal subunits and translation initiation factors, including eIF2α, eIF3η, and eIF5 whose expression is altered in AD, in some cases early in the disease [126, 159]. Decreased synthesis of ribosomal proteins RPL23, RPLP0, RPL19, and RPS16 is also observed in mouse models of tauopathy [78].

Ribosomal subunits were identified as modifiers of Aβ toxicity in *C. elegans* and *S. cerevisiae*. Deletion or knockdown of the orthologs of human *RPL8*, *RPS13*, *RPS16*, and *RPS19* suppressed Aβ toxicity in both yeast and worm models. However, decreased toxicity in response to a reduction of ribosomal protein expression is difficult to interpret. While these interactions may be relevant, it is also possible that they act by reducing the expression of the transgenes used to induce toxicity. Deciphering the role of ribosomal proteins therefore presents a challenge with these models.

While there is the potential for a reduced rate of translation to be protective in neurodegeneration, several ribosomal proteins have extra-ribosomal functions that may be relevant to neurodegeneration. Some ribosomal proteins interact with MDM2 preventing its interaction with, and degradation of p53. No MDM2 ortholog has been identified in yeast or *C. elegans*, suggesting this may not be a function of ribosomal proteins in these organisms. *RPL9* knockdown is associated with Id-1/NF-κB signaling inactivation [10]. Enhanced NF-κB activation is observed in AD patients and is believed to contribute to disease pathology [147, 286], suggesting *RPL9* reduction may be protective by reducing NF-kB signaling. Similarly, RPL11 inhibits PPARα activity, whose activation is neuroprotective [109, 322]. RPL26 enhances p53 translation [40, 289], whose expression is increased and plays a critical role in AD [37, 143, 207]. Defining how specific ribosomal protein genes regulate AD will prove challenging but may identify new therapeutic targets.

### Protein homeostasis is required to prevent neurodegeneration

Impaired protein homeostasis is a characteristic of many neurodegenerative disorders, including AD [45, 329]. The excessive burden of protein misfolding triggers ER stress and activates the unfolded protein response (UPR), a conserved signaling pathway that increases ER folding capacity and inhibits new protein synthesis [128]. In the short term, activating the UPR increases the expression of ER chaperones and helps maintain protein homeostasis. However, prolonged UPR activation can provoke apoptosis [92]. Genome-wide expression analysis revealed that, as in mammalian models, Aβ induces ER stress and activates the UPR in yeast and *C. elegans* [41, 122].

In yeast, UPR response is mediated by the IRE1α branch of the UPR^ER^ that activates the transcription factor XBP1s; it is the only conserved branch in yeast. In a yeast model expressing Aβ_42_, compounds that inhibit UPR prevent apoptosis and confer a protective effect [58]. In *C. elegans*, knockdown of *xbp-1* reduces Aβ aggregation and delays paralysis in the Aβ_42_ model [260], suggesting a negative impact of UPR activation in this model. However, in a *C. elegans* Tau model, loss of *xbp-1* function exacerbated Tau toxicity and constitutive activation of XBP-1 promoted the clearance of misfolded Tau and attenuated Tau-related phenotypes [311]. These seemingly disparate findings may be distinguished by their effects on the long-term and short-term consequences of UPR activation or may reflect differences in the model used or the cell type expression of the transgenes.

Overall, UPR activation may either promote or prevent neurodegeneration, depending on the stages of the disease and specific branches of the pathway affected [127]. The UPR effectors PERK and downstream eIF2α are activated in human AD patients, where they co-localize with abnormal Tau protein [304]. Interestingly, two drugs that inhibit eIF2α activation, trazodone and dibenzoylmethane, are neuroprotective in mouse models of dementia [116].

### Protein degradation pathways prevent the accumulation of misfolded protein

Proteostasis is maintained by the regulation of protein synthesis and degradation pathways. Misfolded or toxic proteins are prevented from accumulating by the two protein degradation systems, the ubiquitin-proteosome system (UPS) and autophagy [106, 210, 226]. In addition to its role in protein degradation, autophagy is involved in the extracellular release of Aβ and plaque formation [221, 222]. Dysregulation of the ubiquitin–proteasome system is observed in patient samples [226]. In neurodegenerative disorders, dysfunctions of protein degradation pathways have been identified as contributors to neurodegeneration [301, 302]. This has also been demonstrated in *C. elegans, Drosophila*, and yeast models of AD (Tables S11-S14). The *C. elegans* AIRAP/AIRAPL homolog AIP-1, a component of the proteasome 19S regulatory cap, plays an essential role in preventing AD phenotypes in worms [123]. *Drosophila mir-9a* enhances Tau-related phenotypes by repressing the UBE4B ubiquitin ligase that targets Tau for degradation [285]. Overexpression of Atg, the *Drosophila* ortholog of ULK1, a mediator of autophagy, suppressed Aβ toxicity [30]. It is likely that the *C. elegans* ortholog of this gene, *unc-51*, was not identified in RNAi screens designed to identify enhancers of neurodegeneration because RNAi clones that produce an uncoordinated phenotype on their own are generally excluded from consideration.

In many models of neurodegeneration, increasing the activity of protein degradation pathways can reduce neurodegeneration [236, 251]. For example, in *Drosophila* deficiency of S5b/PSMD5, the 26S proteasome regulatory subunit increases proteasome activity and reduces Tau rough eye phenotype [271]. Another AD-relevant protein, CD2AP, is vital for the UPS in a *Drosophila* AD model [229]. CD2AP mutation inhibits proteasome activity and synaptic vesicle recycling, which enhances Tau neurotoxicity in flies.

### Mitochondrial dysfunction in AD

The central nervous system has high energy demands; although it represents 2% of the body’s weight, it consumes 20% of the total oxygen [149, 276]. This energy is provided by mitochondria, which are essential for ATP and amino acid production and maintaining calcium homeostasis [233, 256]. Mitochondrial dysfunction is a hallmark of AD. Defects in mitochondrial morphology, dynamics, trafficking, and mitophagy occur in AD [250, 283, 314]. Such dysfunction leads to increased ROS, decreased ATP production and altered ion homeostasis [36, 59, 212, 213, 279, 287]. These phenotypes are also observed in AD models, suggesting that the involvement of mitochondrial dysfunction is conserved across species.

Both Aβ and Tau models produce mitochondrial pathologies. Firstly, Aβ accumulates in the mitochondria of AD patients and in Aβ transgenic mouse, yeast, and *Drosophila* models (Anandatheerthavarada et al. 2003, [34, 43, 134, 194, 234, 288]). As previously discussed, DNAJ proteins have been implicated in the transport of Aβ into the mitochondria. In addition, a recent study in yeast showed that Aβ is specifically recognized by the mitochondrial translocase of outer mitochondrial membrane subunit 22 (TOMM22) and that Aβ transport into the mitochondria depends on the TOM complex [134]. Another component of the TOM complex, TOMM40 is a susceptibility gene for late-onset AD [112]. Overexpression of TOMM22 or TOMM40 increased mitochondrial Aβ in a human cell line and was accompanied by changes in mitochondrial morphology, mitochondrial damage and an increase in autophagosomes and autolysosomes [65].

Mitochondrial dysfunction may also occur when cells are unable to eliminate damaged mitochondria. In *C. elegans*, expression of disease-associated Tau (P301L) inhibits mitophagy [54]. For neuronal cells, the location of mitochondria is an added consideration. In *Drosophila*, axonal loss of mitochondria enhances neurodegeneration in a Tau-based model [140]. Moreover, expression of disease-associated mutant forms of Tau enhanced mitochondrial elongation in both *Drosophila* and mouse models. Increasing mitochondrial fission reduced mitochondrial length and neurotoxicity in *Drosophila*, suggesting that abnormal mitochondrial dynamics promote neurodegeneration [70].

In high throughput studies, many mitochondria-related genes were identified as modifiers of AD phenotypes. Notably, in *C. elegans,* RNAi-mediated knockdown of electron transport chain components, including the ATP synthase subunits *atp-2* and *atp-5* (complex V) and the complex I NADH ubiquinone oxidoreductase *nuo-2*/NDUFS3, and NADH dehydrogenase *nuo-3*/NDUFA6, suppressed paralysis in Aβ_42_ models, although the mechanisms were unclear [154]. One possibility is that partial knockdown generates a mild mitochondrial stress that induces a protective response. In both *C. elegans* and *Drosophila*, a slight decrease in the activity of the mitochondrial respiratory chain increases lifespan [49, 83, 117, 176]. A severe reduction of ETC function is lethal in *C. elegans* [300].

### Regulation of oxidative stress in AD

Oxidative damage is an early event in AD development, contributing to toxic oligomer formation and disease development [125, 196, 205, 224, 235, 309]. Oxidative stress causes DNA damage [266], protein oxidation [14], lipid peroxidation [220], contributes to neuronal cell damage, and promotes apoptosis [187, 249].

As in AD, oxidative stress contributes to toxicity and neurodegeneration in model systems [67, 312]. High throughput screens conducted in yeast identified 25 Aβ modifiers involved in oxidative stress response (Tables S9, S10). Similarly, a high throughput genetic modifier screen in a *Drosophila* model of AD identified targets significantly enriched in oxidative stress-related genes [255]. This study also showed that overexpressing antioxidative genes, specifically genes encoding the iron-binding protein ferritin and H_2_O_2_ scavenger catalases suppressed Aβ toxicity. Furthermore, knocking down *mitoferrin-1*, a mitochondrial iron transporter, reduced ROS and extended lifespan in *C. elegans* AD models, indicating its critical role in regulating mitochondrial iron metabolism in AD [136]. Interestingly, a mild increase in ROS can be neuroprotective by the formation of glial lipid droplets that transfer peroxidized lipids from neurons to glia, where homologs of AD-risk genes *ABCA1*, *ABCA7*, *VLDLR*, *VPS26*, *VPS35*, *AP2A*, *PICALM*, and *CD2AP* are required in *Drosophila* [214].

The association between oxidative stress and Tau phosphorylation is controversial. Treatment of H_2_O_2_ leads to decreased Tau phosphorylation in rat hippocampal and SH-SY5Y human neuroblastoma cells [331], but chronic oxidative stress through inhibition of glutathione synthesis increased Tau phosphorylation in M17 neuroblastoma cells [284]. This increased phosphorylation is proposed to occur as a result of increased activity of JNK and p38 MAPK and decreased activity of PP2A [284]. Vanhelmont and colleagues reported that oxidative stress induces Tau aggregation in yeast but decreases Tau phosphorylation [308]. Similarly, in *Drosophila*, increased oxidative stress increases neurodegeneration, but not by increasing Tau phosphorylation [60]. Thus, Tau phosphorylation and oxidative stress may work in parallel to promote aggregation.

### Cellular trafficking influences Aβ toxicity

Defects in cytoskeletal dynamics, vesicle trafficking and sorting systems are observed in AD [180]. Genes related to cellular trafficking were recovered in yeast and *C. elegans* screens. In yeast, Aβ expression impairs clathrin-mediated endocytosis [297]. Single nucleotide polymorphisms in phosphatidylinositol binding clathrin assembly protein (PICALM), an adapter protein that functions in clathrin-mediated endocytosis and autophagy, are associated with AD [119, 172, 327]. PICALM has been implicated in the trafficking and processing of APP, the turnover of Aβ, and as a modulator of glutamatergic signaling [294, 325, 331]. Overexpression of PICALM orthologs in *C. elegans* and *Drosophila* protect against neuronally expressed Aβ [238, 297, 330]. Surprisingly, PICALM increased Aβ toxicity in a yeast model expressing an ER-targeted Aβ_42_-GFP fusion protein [55] and in *C. elegans*, the knockdown of the PICALM ortholog *unc-11* suppressed Aβ toxicity when Aβ_42_ was expressed in the muscle [215]. While these data are taken from large-scale screens that require additional validation, these seemingly contradictory findings may be the result of the many roles of PICALM and further investigation is needed to disentangle these effects.

Defects in ER-Golgi trafficking reduce Aβ toxicity in yeast, whereas mutations in genes involved in cytoskeleton, endocytosis, and the ESCRT machinery which function in vesicular trafficking, increase Aβ toxicity [93]. However, in *C. elegans*, endocytic gene depletion suppresses necrotic neurodegeneration [299]. In addition, a large-scale RNAi screen in *C. elegans* identified several genes involved in ER to Golgi trafficking, including *copd-1*, *sly-1*, *syx-5*, *sec-12, sec-23*, *and rab-1,* that when knocked down suppress Aβ toxicity [154].

In both *C. elegans* and *Drosophila* screens, cytoskeletal proteins and regulators modified outcomes in Tau-based models (Tables S2, S3). These regulators comprise a range of proteins that bind or modify actin or microtubules.

Notably, genes involved in F-actin processing were identified in screens in both organisms. F-actin can associate with Tau [94], which may mechanistically explain how these proteins act as modifiers in Tau-based models. In general, F-actin-associated proteins promoted worse outcomes. This is consistent with the finding in *Drosophila* that overexpression of actin (Act5C) exacerbates toxicity resulting from overexpression of human Tau [94]. Likewise, knockdown of the *C. elegans* actins, *act-1* and *act-5*, suppresses paralysis in an Aβ_42_ model [154].

### Lipid metabolism influences neurodegeneration

Dysregulation of glucose and lipid metabolism have been implicated in the development of AD [185, 328]. In *C. elegans,* the knockdown of four genes involved in fatty acid biosynthesis, *elo-4*, *acs-1*, *fat-6*, and *pod-2,* suppressed paralysis in an Aβ model. Increased expression of the orthologs of these four genes *ELOVL3*, *ACSF2*, *SCD5*, and *ACACA* was reported in either mouse models or AD patients [8, 11, 184, 240], suggesting they may have conserved roles in AD. Similarly, decreased expression of the Δ9 desaturases *fat-5* or *fat-7* rescues neurodegeneration in a *C. elegans* model of Parkinson’s disease [201]. Together these data suggest that specific lipids may either promote or protect against neurodegeneration. Consistent with an important role for lipid metabolism in AD, Triggering Receptor Expressed on Myeloid Cells 2 (TREM2) and ATP-binding cassette transporter A7 (ABCA7), two genes with risk variants associated with AD function are required for lipid homeostasis [148, 282].

### Cell signaling in AD

Many conserved cell signaling pathways can influence AD development [103, 129] including Wnt, MAPK, and TOR pathways [227, 313, 338].

Wnt signaling pathway components were identified in both *C. elegans* and *Drosophila* screens [165, 275]. GSK-3β phosphorylates Tau at several disease-relevant sites [118, 189, 191], but also antagonizes the Wnt pathway, both functions may be relevant to neurodegeneration. In *C. elegans*, knocking down *gsk-3* enhances Tau-related neurodegeneration. In *Drosophila*, downstream mediators of the Wnt pathway have also been identified as modifiers. Overexpression of the *Drosophila* β-catenin exacerbates neurodegeneration in a Tau model [142].

Gain-of-function mutations in the fly *TAOK1* homolog enhanced disease phenotype in a *Drosophila* model of tauopathy [274]. The thousand-and-one kinases (TAOKs) belong to the MAP3K family. TAOKs have many targets, including proteins in the p38 and Hippo pathways [81]. Furthermore, a TAOK inhibitor reduces Tau phosphorylation in mice and induced pluripotent stem cell-derived neurons from frontotemporal lobar degeneration (FTLD) patients [99].

**Fig. 6:**
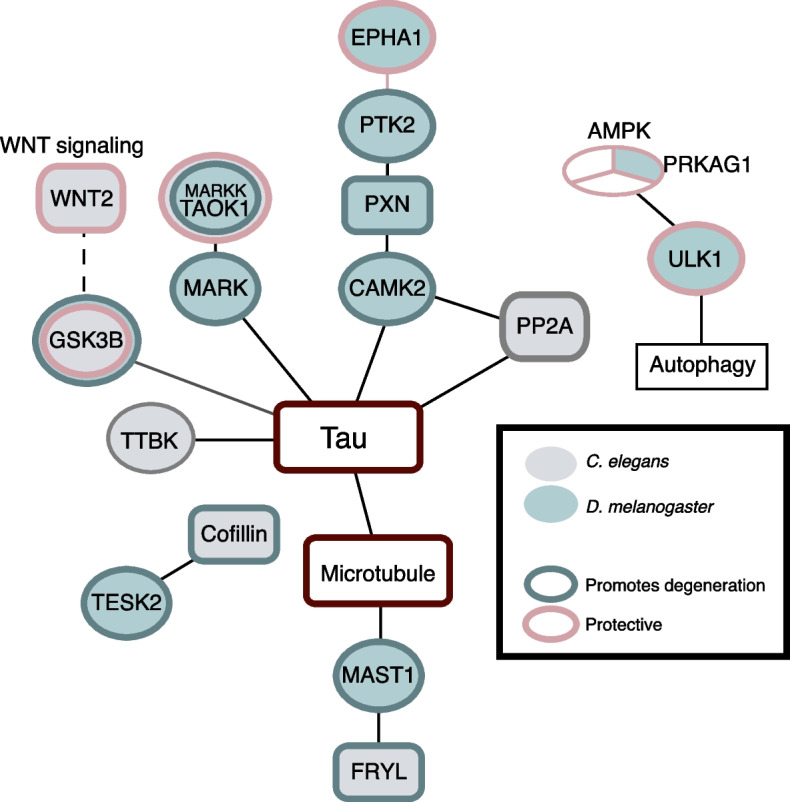
Tau related kinases recovered in *C. elegans* and *Drosophila* AD modifier screens. Fill colour indicates species, line colour indicates effect on neurodegeneration. Nodes are connected by phosphorylation or signaling events. Nodes with 2-colour outlines indicate genes where findings between studies are incongruent

Tau is heavily phosphorylated in AD, and these modifications are believed to contribute to the disease. Many kinases that phosphorylate Tau have been identified, and many are conserved in *C. elegans* and *Drosophila*. Further, when human Tau is expressed in *Drosophila* or *C. elegans*, it is phosphorylated at disease-relevant sites [165, 248]. While there is limited overlap at the single gene level, orthologs of kinases that phosphorylate human hTau were identified as modifiers in both *C. elegans* (TTBK, TAOK, GSK-3) and *Drosophila* (CaMKII, MARK, TAOK, GSK-3) (Fig. 6, Supplementary Tables 3, 4). The ability of these proteins to phosphorylate Tau may be conserved across organisms. Consistent with this idea, many phosphorylation sites, including SKXGS sites and several proline-directed serines, are conserved in *Drosophila* and *C. elegans* Tau proteins. Nevertheless, there are likely species-specific ways in which Tau is modified and regulated. Intriguingly, in *Drosophila*, the activity of these genes correlates with increased phosphorylation having a negative impact. By contrast, In *C. elegans*, knockdown of the orthologs of GSK-3β, TAOK and TTBK enhanced toxicity, contrary to what is expected based on their abilities to phosphorylate Tau.

Downregulation of the focal adhesion kinase (Fak), the *PTK2B* homolog, suppressed Tau toxicity in a fly model [66]. PTK2B co-localized with hyperphosphorylated Tau in AD patient brain samples, suggesting that it may directly phosphorylate Tau. PTK2B was also implicated in Aβ regulation, but the mechanisms involved remain elusive, as two different transgenic mouse models found seemingly opposing roles for the protein, with PTK2B deletion or overexpression were both shown to be protective [101, 263].

### Challenges and limits to the use of model systems in Alzheimer’s research

While simple model organisms provide many advantages in discovery research, differences in the biology of humans and model organisms can provide a challenge in modelling certain aspects of the disease. For example, neuropsychiatric symptoms in AD cannot be modelled in simple organisms. Specific anatomical and biological differences between humans and model organisms may also limit the study of some aspects of the disease. *Drosophila* and *C. elegans* do not accumulate neurofibrillary tangles [320, 321]. Further, microglia play an important role in the progression of AD, contributing to phagocytosis and inflammation [208] but *C. elegans* and *Drosophila* do not have an obvious equivalent cell type. Although it is possible that some roles of the microglia are filled by other cells, some genes that play important roles in AD are highly expressed in microglia. Many proteins believed to influence AD through their activity in the microglia, including TREM2, CLU and CD33, do not have orthologs in *Drosophila* or *C. elegans*.

Differences in the biology of humans and model organisms can be an advantage and a disadvantage. In the case of neurofibrillary tangles, the observation that neurodegeneration occurs in the absence of neurofibrillary tangle formation was an important demonstration that toxic forms of Tau precede neurofibrillary tangle formation [320]. Similarly, the absence of an adaptive immune system facilitates the examination of processes independent of inflammation.

The use of overexpression models may be a limitation in that expression is generally much higher than what would be observed in AD. Also, the ability of transgenes to promote rapid degeneration may not model all aspects of a disease that progresses more slowly. These effects may bias which genes are recovered as modifiers. Differences in levels of expression of different transgenes could explain the limited overlap between screens where the same protein is being expressed. The timing and location of gene expression could also explain differences between screens. Expression of these transgenes during development could induce developmental effects that predispose animals to more rapid aging or decreased stress response. Moreover, simple overexpression models cannot capture subtleties in the production of Aβ or Tau. Aβ overexpression models typically use the Aβ42 peptide alone and therefore do not integrate the regulation of APP cleavage or the potential influence of other forms of Aβ. Similarly, Tau-based models typically overexpress one of the six Tau isoforms normally found in the human brain. The specific isoform chosen, as well as the absence of multiple isoforms, may affect which modifiers are isolated in a given screen.

The design of individual models and screens affects which genes are recovered and which are not. An advantage of *Drosophila* and yeast is the ability to easily perform both gain-of-function and loss-of-function screens, whereas resources for large-scale gain-of-function screens in *C. elegans* are lacking. Modifiers identified by overexpression in *Drosophila* may not be identified in *C. elegans* because their impact is not obvious in knockdown experiments. For amyloid and Tau-based screens, the cell type chosen for expression may also impact which genes are identified as modifiers. In *C. elegans*, early screens used a muscle-expressed Aβ, Tau models on the other hand, used neuronal expression.

Species-specific gene function may explain why some gene modifiers do not translate directly to human biology and genes that act as modifiers in humans may not function as such in model organisms. There are several reasons a human risk gene might not be uncovered as a modifier in a model organism. Large-scale screens in these organisms often rely on knockdown or knockout approaches, while disease-associated alleles may produce more subtle effects, for example, an amino acid substitution that produces a specific functional alteration. Species-specific expansions of gene families may create redundancy that masks the function of individual genes. Functions specific to either humans or the model system may also explain the limited overlap in screens. In some cases, an ortholog to a human disease gene may not exist, for example, APOE, the strongest risk factor for late-onset AD [183], does not have a direct ortholog in *Drosophila* or *C. elegans*. In other cases, the functional ortholog may not have been identified. In human disease-associated genes identified in GWAS studies and genes identified in genetic screens, there is overlap in protein families that is not captured when analyzing on a gene by gene basis. For example, DNAJ family proteins have been identified in GWAS studies and as modifiers in all three organisms examined, but they do not overlap on a gene-by-gene basis using predicted orthologs.

## Summary

The study of human AD samples has provided a wealth of information; however, it remains a challenge to decipher cause from consequence using only these samples. Model organisms allow rapid hypothesis testing and unbiased genetic screening that contribute to the discovery of AD-related cellular processes and signaling pathways. Integration of data across different models can be a powerful approach to understanding the biology of neurodegeneration.

AD modifiers identified from high throughput screens and targeted studies can be classified into functional categories relevant to neurodegeneration. Major pathways involving Aβ modifiers identified in all three model systems examined include transcription and translation-related processes, stress response and chaperones, and protein trafficking. When comparing Tau modifiers found in *C. elegans* and *Drosophila*, transcription and translation-related processes, stress response and chaperones, cytoskeleton-related pathways, and metabolism are shown to play critical roles in regulating abnormal Tau expression. These evolutionarily conserved pathways reveal fundamental mechanisms of AD and other neurodegenerative disorders.

Caution should be taken in interpreting negative findings from large-scale screens. These screens are generally designed for ease of screening and can be biased toward dramatic effects, while missing more subtle ones. In addition, whether a gene was effectively interrogated in a given screen depends on whether it is present in a deletion set or RNAi library, whether loss of function is lethal or produces a phenotype that excludes it from consideration, and whether it is expressed in the cell type and at the age examined. For example, in *C. elegans*, knockdowns that produce a movement defect, or incoordination in a wild-type background, are generally excluded from analysis when a screen measures the enhancement of movement defects. Additionally, the presence of paralogs or other redundantly functioning genes can hide the involvement of some genes in neurodegeneration. Species-specific gene duplications could therefore result in a gene being recovered in a screen in one organism, but not in another.

Caution should also be exercised in interpreting positive results from large-scale screens without additional validation. While we have highlighted some findings from these screens, many require additional validation, including the analysis of mutants and more direct analysis of neurons, rather than a phenotypic proxy. Determining whether effects observed are cell autonomous or non-cell autonomous can also clarify the role of a specific modifier in neurodegeneration. While the ability to perform screens in whole animals is an advantage, it is important to consider that modifiers recovered may not function cell-autonomously.

Some genetic modifiers identified in model organisms do not have obvious human orthologs and their functions remain unknown. These genes may represent species-specific signaling or these genes may have human orthologs that cannot be identified on the basis of sequence homology. Nevertheless, their activities may be related to processes that also influence neurodegeneration in humans.

The use of simple model systems to study AD and related tauopathies has revealed important cellular mechanisms of neurodegeneration and provides powerful tools for discovering therapeutic targets and strategies to combat these diseases. In combination with cell lines, animal models, and clinical studies, simple model organisms can provide insights into disease mechanisms and aid in the development of effective treatments for AD and other neurodegenerative disorders. In fact, studies that combine different models can be very powerful (Kim et al., 2019). These studies leverage the conservation of processes between animals to identify robust modifiers.

### Supplementary Information

**Additional file 1:** **Table S1.** Genetic modifiers of AD identified in *S. cerevisiae* models. **Table S2.** Genetic modifiers of AD identified in *C. elegans *models. **Table S3.** Genetic modifiers of AD identified in *Drosophila* models. **Table S4.** Human orthologs of genetic modifiers identified in Aβ models. Human orthologs of *S. cerevisiae* genes were retrieved from the *Saccharomyces* Genome Database (SGD) YeastMine online tool. Orthologs of *C. elegans* genes were queried from the OrthoList2 online tool. Orthologs of *Drosophila* genes were obtained from FlyBase. AD genes identified from GWAS studies are highlighted in red. **Table S5.** Human orthologs of genetic modifiers identified in Tau models. Orthologs of *S. cerevisiae* genes were retrieved from the SGD YeastMine online tool. Orthologs of *C. elegans* genes were queried from the OrthoList2 online tool. Orthologs of *Drosophila* genes were obtained from FlyBase. AD genes identified from GWAS studies are highlighted in red. **Table S6.** Functional classes enrichment of Aβ modifiers identified in *S. cerevisiae* models analyzed using FunSpec. Gene list was queried to multiple yeast databases including GO Molecular Function, GO Biological Process, GO Cellular Component, MIPS Functional Classification, MIPS Phenotypes, MIPS Subcellular Localization, MIPS Protein Complexes using FunSpec. **Table S7.** Cellular pathways and processes linked to Tau modifiers. GO analysis of Tau modifiers identified in *S. cerevisiae* models was performed using FunSpec. Tau modifiers identified in *C.elegans*models were analyzed using the WormCat online tool. Tau modifiers identified in *Drosophila *models were grouped using the GLAD database. **Table S8.** Functional annotation and enrichment of Aβ modifiers identified in *C. elegans* models analyzed using WormCat. Gene list were input to WormCat using default settings for analyzation. Promoting genes indicated that deletion/reduction suppresses AD phenotypes and/or overexpression enhances AD phenotypes. Preventing genes indicated that deletion/reduction enhances AD phenotypes and/or overexpression suppresses AD phenotypes. **Table S9.** Functional annotation and enrichment of Tau modifiers identified in *C. elegans* models analyzed using WormCat. Gene lists were analyzed using WormCat. Promoting genes indicated that deletion/reduction suppresses AD phenotypes and/or overexpression enhances AD phenotypes. Preventing genes indicated that deletion/reduction enhances AD phenotypes and/or overexpression suppresses AD phenotypes. **Table S10.** Functional classes enrichment of Aβ modifiers identified in *S. cerevisiae* models analyzed using GO Slim Mapper. Gene list was queried to SGD Yeast GO-Slim database. **Table S11.** Functional annotation and enrichment of Aβ modifiers identified in *C. elegans* models analyzed using WormCat. Gene list were input to WormCat using default settings for analyzation. Promoting genes indicated that deletion/reduction suppresses AD phenotypes and/or overexpression enhances AD phenotypes. Preventing genes indicated that deletion/reduction enhances AD phenotypes and/or overexpression suppresses AD phenotypes. **Table S12.** Functional annotation and enrichment of Aβ modifiers identified in *Drosophila* models analyzed using GLAD. Gene list was analyzed using Find Group Membership function in the GLAD online tool [5, 6, 12, 19, 26, 28, 31, 33, 38, 39, 44, 47, 48, 52, 56, 57, 61, 68, 71, 73–75, 77, 79, 85, 90, 95–98, 100, 102, 111, 113, 115, 121, 131, 137, 145, 146, 152, 153, 155, 157, 161, 163, 164, 166, 170, 174, 175, 177–179, 181, 186, 188, 190, 192, 197, 199, 200, 202, 203, 211, 217–219, 223, 228, 230, 231, 237, 239, 241, 244, 246, 247, 253, 259, 262, 265, 272, 273, 280, 290, 292, 293, 295, 296, 298, 303, 306, 307, 310, 315, 316, 318, 319, 323, 326, 332–334, 336, 337].
